# Relapsed High-Risk Medulloblastoma: Stable Disease after Two Years of Treatment with Somatostatin Analog - Case Report

**DOI:** 10.7759/cureus.446

**Published:** 2016-01-04

**Authors:** Luisa Galvis, Diego Gonzalez, Carlos Bonilla

**Affiliations:** 1 Oncology, Instituto Nacional de Cancerologia, Bogota, Colombia; 2 Oncology, Instituto de Cancerologia Las Américas, Medellin, Colombia

**Keywords:** medulloblastoma, octreotide, somatostatin

## Abstract

Cerebellar medulloblastoma in adults is an uncommon disease. Therefore, most information comes from the pediatric population, and the treatment for relapses is based on series and case reports. The expression of somatostatin receptors has been identified in most medulloblastoma patients, and preclinical experience has shown a promissory response to somatostatin analogs. This report presents a female patient with a high-risk left cerebellar medulloblastoma diagnosed at age 16 years old who was treated with complete resection, cranial-spinal radiotherapy, and chemotherapy. She presented again at 18 years of age with a sustained progression of her tumor, despite radiosurgery and another line of chemotherapy. Octreotide scintigraphy at that time showed a moderate to high expression of somatostatin receptors; thus, the patient was started on monthly octreotide. She is now 20 and has achieved stable disease over more than two years of active treatment without any drug-related toxicity. Somatostatin analogs could be considered as a treatment option in selected cases of medulloblastoma. Review of the literature is presented for this unusual response.

## Introduction

The standard treatment for adult medulloblastoma patients consists of surgical resection followed by adjuvant radiotherapy and, in some cases, chemotherapy [1]. There is no standard chemotherapy regimen, but several combinations have been used, including vinca alkaloids, platinum compounds, etoposide, anthracyclines, among others. There is no standard algorithm for the treatment of patients who relapse or progress after first-line chemotherapy. Since it has been shown that these tumors may have a high expression of somatostatin receptors, especially subtype 2 receptors (SSTR2), somatostatin analogs, such as octreotide, could have a role in the management of this disease. However, there is not enough evidence thus far to support this approach.

## Case presentation

**Figure 1:**
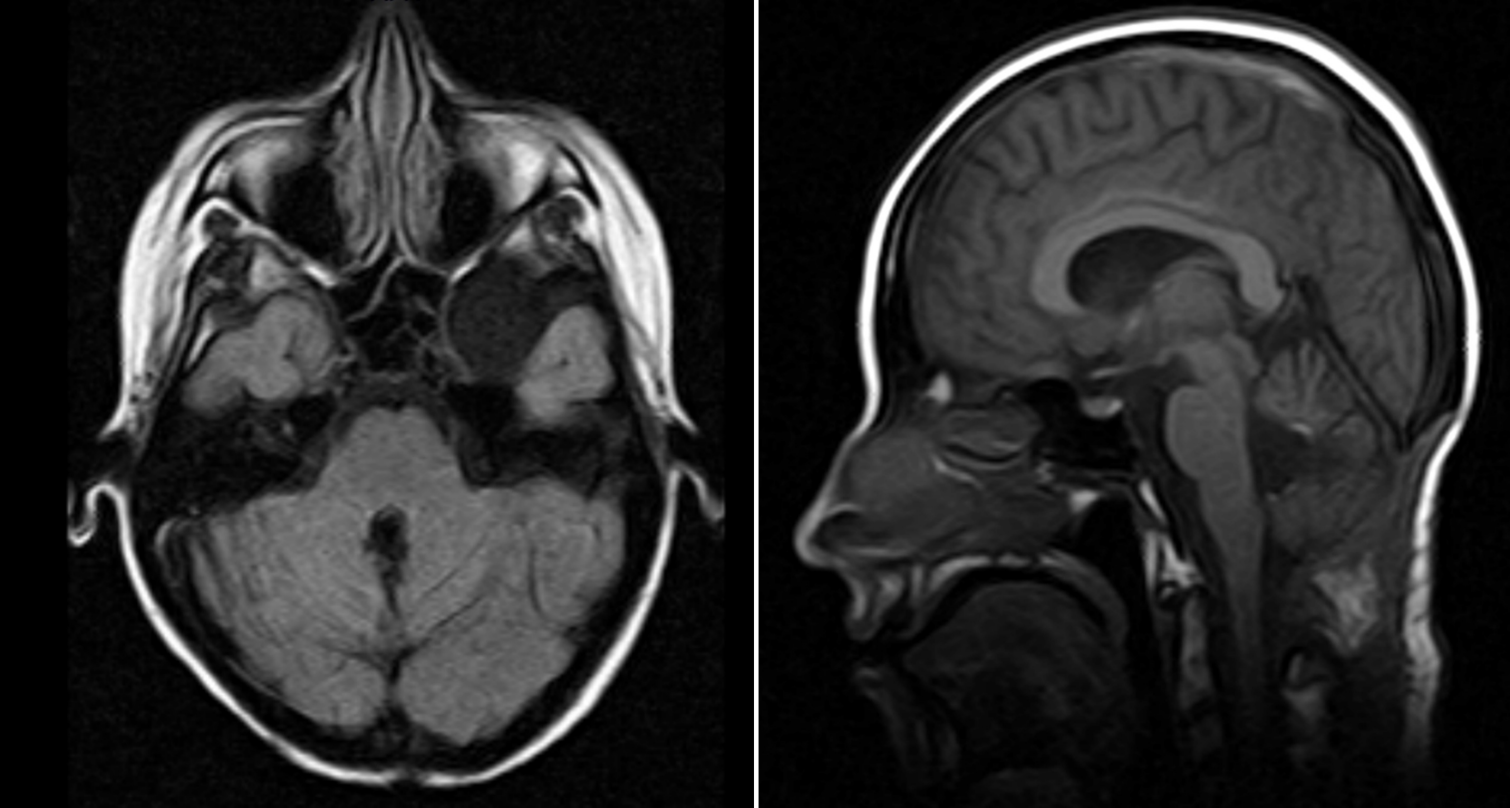
Postoperative magnetic resonance imaging (MRI) in February 2011 Left: Axial view Right: Sagittal view

This female patient presented in 2010, at the age of 16 years, with an intense headache, dysarthria, dysmetria, and gait disturbances. She was found to have hypotonia and nystagmus in the initial consult and, therefore, manifested an overt cerebellar syndrome. Informed patient consent was obtained. Brain imaging done at that time showed a round mass arising from the vermis in the cerebellum, protruding into the fourth ventricle. She underwent optimal resection in February 2011, and the pathology specimen confirmed cerebellar medulloblastoma. Her postoperative magnetic resonance imaging (MRI) is shown in Figure 1.

**Figure 2:**
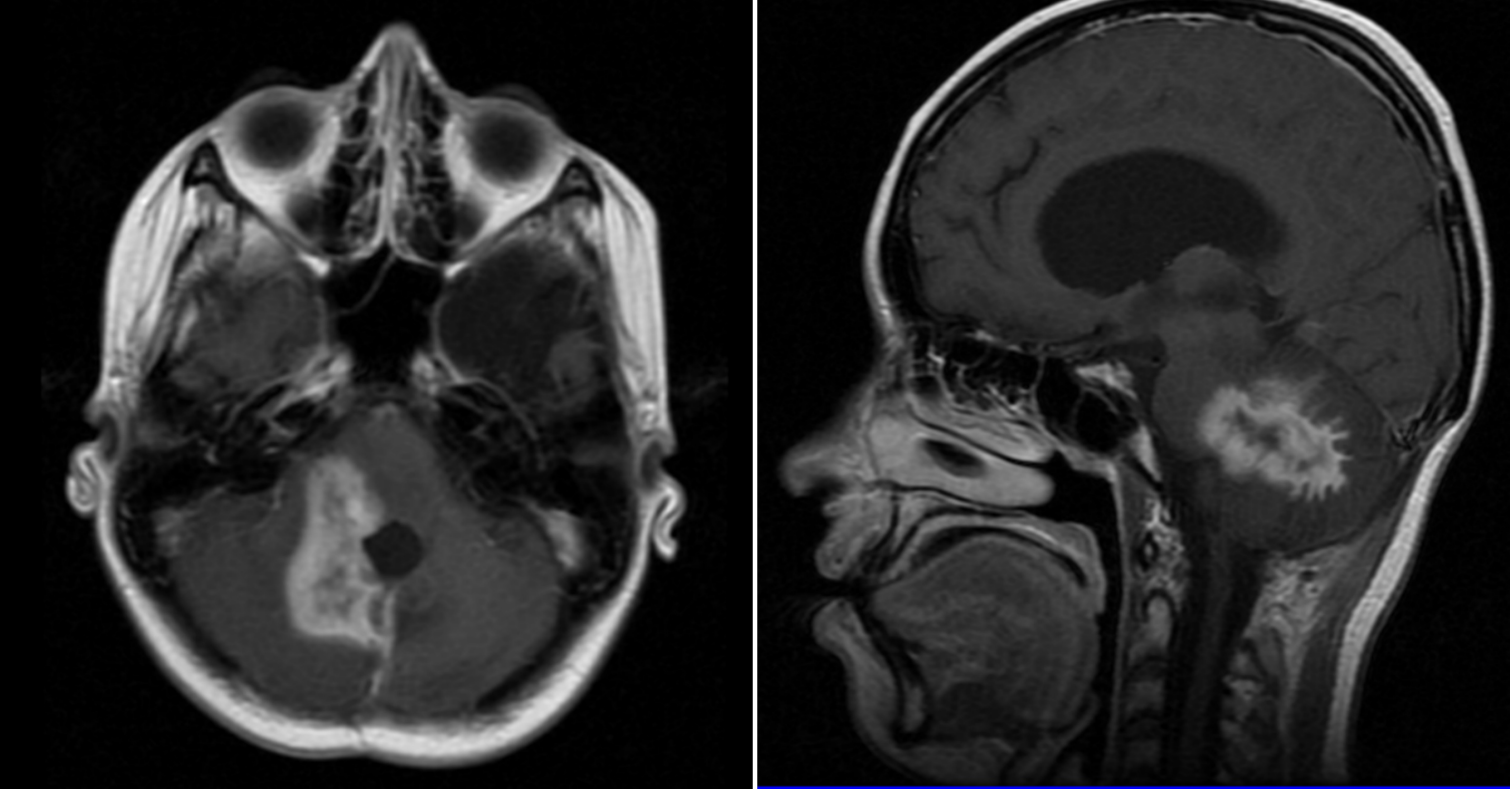
Magnetic resonance imaging (MRI) showing cerebellar vermis progression in November 2012 Left: Axial view Right: Sagittal view

Because of the high-risk characteristics, she received adjuvant cranial-spinal radiation and sequential adjuvant chemotherapy with cisplatin, etoposide, and cyclophosphamide for six cycles, completed in October 2011. Seven months after ending the treatment, a cerebellar relapse in vermis was documented, requiring radiosurgery and temozolomide for four months, when a new progression in the same location was documented (Figure 2).

By the time of the second relapse, the patient had severe ataxia, remarkable neurocognitive impairment, and moderate to severe functional dependence secondary to pronounced cerebellar dysfunction symptoms. Given the rapid progression to radiotherapy and two lines of chemotherapy, as well as the toxicity with previous treatment and neurological sequela, new cytotoxic therapy was not considered.

**Figure 3:**
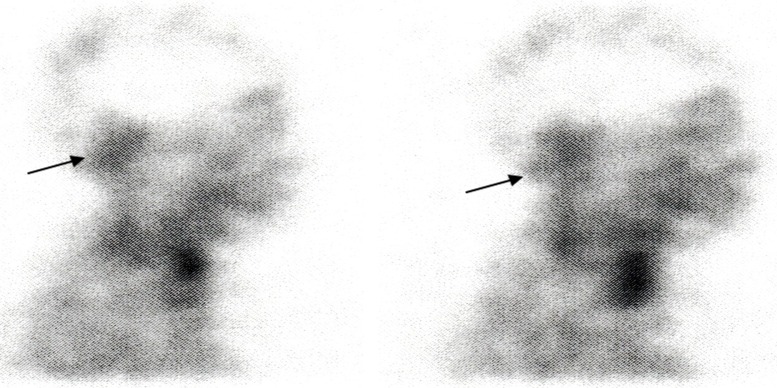
In-111 DTPA octreotide scintigraphy in March 2013 showing moderate expression of somatostatin receptors Left: 4 hours after infusion Right: 24 hours after infusion

In an attempt to search for other treatments, a scintigraphy with octreotide was performed, which showed moderate expression of somatostatin receptors, as can be seen in Figure 3.

**Figure 4:**
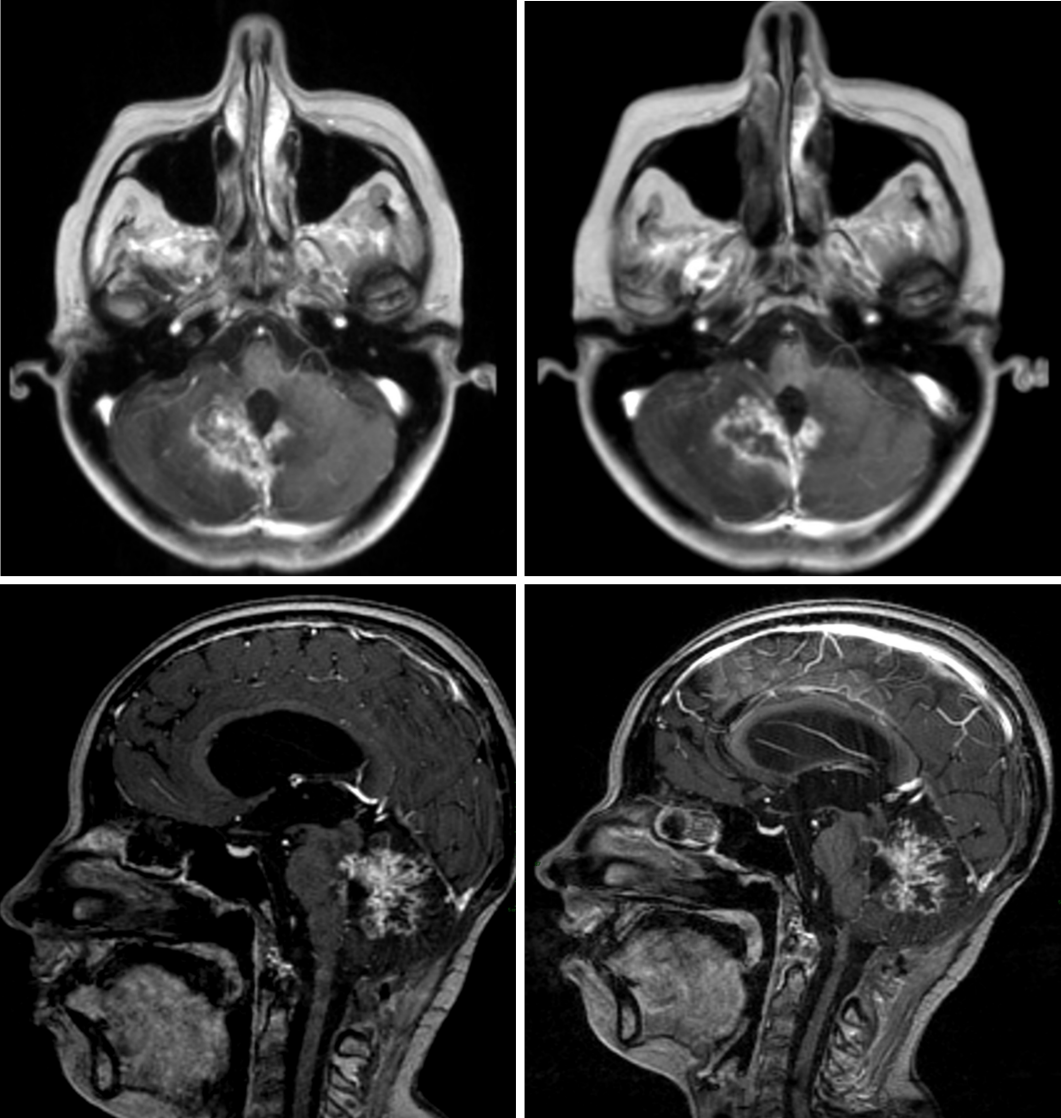
Stable disease in magnetic resonance imaging (MRI) during octreotide treatment Left (Top and Bottom): Axial and sagittal view in February 2014 Right (Top and Bottom): Axial and sagittal view in May 2015

The patient was started on monthly intramuscular injections of octreotide LAR (long-acting release), 30 mg per dose, beginning in May 2013. By May 2015, she had undergone 24 months of treatment with a stable lesion on imaging (Figure 4) and clinical improvement, partially recovering independence and functionality, without any drug-related toxicity.

## Discussion

Medulloblastoma is an aggressive embryonal tumor of the cerebellum that can occur at any age. It has a peak incidence between three and seven years. Medulloblastoma is the most common tumor of the central nervous system (CNS) in children (15-30% of cases) while in adults it represents only 3% of primary CNS tumors [1]. This neoplasia has a predilection to spread through the cerebrospinal fluid anywhere in the central nervous system and other extra-CNS locations in advanced stages [1].

The most widely accepted staging system was proposed by Chang [2], including tumor size, the involvement of neighboring or external structures, and cerebrospinal fluid. Patients can be classified as average or high-risk, depending on the presence of metastasis, postoperative residual tumor greater than 1.5 cm^2^, and anaplastic or large cell histology [3]. The histological subtype is also considered in the 2007 World Health Organization (WHO) CNS classification [1]; it has been proposed recently to include genomic characteristics in the risk stratification: hedgehog pathway activation, Wnt, and c-myc [3] 

The standard treatment is surgery followed by cranial-spinal radiotherapy. Adjuvant chemotherapy, although frequently used, has not shown consistent results through all studies, especially in patients with average risk; in high-risk patients, it seems to be of benefit [1-2, 4]. There is no universal consensus about the best adjuvant chemotherapy regimen [1]; in adults, one of the most commonly used is a regimen including cisplatin, etoposide, and cyclophosphamide [4].

The treatment of recurrences must be individualized according to the location, extent of relapse, and patient functional status and may include a second resection, re-irradiation, and systemic therapy (chemotherapy, bone marrow transplantation, and immunotherapy) [1-2]. There is no standard chemotherapy regimen for the treatment of relapse, but there are case reports describing the use of irinotecan and bevacizumab, thiotepa with or without carboplatin, ifosfamide, cyclophosphamide, nitrosoureas, vincristine, methotrexate, temozolomide, anthracyclines, procarbazine [5], and vismodegib [6].

The expression of somatostatin receptors has been reported in normal tissues (neurons, glia, pituitary, thyroid, spleen, liver, adrenal, etc.) and also in several malignancies. Medulloblastoma shows a high density of somatostatin receptors, especially Type 2 receptors (SSTR2) [7], which allows scintigraphy with somatostatin or its analogs to be used for initial diagnosis or relapse and to consider the use of therapies directed to these receptors.

Activation of the SSTR2 regulates cellular adenosine triphosphate (ATP) and nucleic acid synthesis, reduces the metabolic activity of the cell, modulates the activity of the MAP kinase (MAPK) pathway, and can even inhibit mitosis and promote apoptosis as demonstrated *in vitro* and in animal models [8].

The clinical utility of somatostatin analogs in the treatment of medulloblastoma was first reported in 2005 in a Serbian series, which presented 10 of 14 children (72%) with a high-risk of relapsing medulloblastoma having disease involution with the use of octreotide alone [9]. In adults, the previous clinical experience is reduced to a single case reported in 2008 by Glas, et al. with a 43-year old man with a relapsing medulloblastoma who achieved sustained disease response with octreotide; unfortunately, he presented with thrombotic thrombocytopenic purpura by the tenth month of administration and, therefore, the treatment was ended [10].

Since all previous treatment options had been exhausted and with the positive results published with the use of octreotide, the oncology team at our institution felt this alternative treatment was a viable option in this difficult case of a young adult with rapid clinical deterioration secondary to progressive relapsed medulloblastoma. As there were no other treatment modalities to offer the patient, the oncology team recommended using octreotide. The family and the patient agreed to try this regimen with a very surprising outcome.

## Conclusions

This paper presents a young adult patient with an extensive relapsed high-risk cerebellar medulloblastoma after receiving cranial-spinal radiation therapy and first-line chemotherapy for six cycles of cisplatin, etoposide, and cyclophosphamide. Following a second rapid progression after radiosurgery and sequential temozolomide, a regimen of monthly octreotide was started. 

To the best of our knowledge, this is the first adult patient to have sustained stable disease using the monthly octreotide, now with over more than 24 months of treatment, without any drug-related toxicity. We believe this is a promising approach to further knowledge in the biology of this tumor and its treatment, especially in those heavily treated patients in whom the balance between potential benefits and harm of medication to be given is a real challenge.
